# Supplementary material for: Comparison of the Effects of High Pressure Processing, Pasteurization and High Temperature Short Time on the Physicochemical Attributes, Nutritional Quality, Aroma Profile and Sensory Characteristics of Passion Fruit Purée
Source: Foods. 2022 Feb 22;11(5):632. doi: 10.3390/foods11050632 (PMC8909329; doi:10.3390/foods11050632)
Supplement: Supplementary file 1 [file foods-11-00632-s001.zip › foods-1536992-supplementary.pdf]

Supplementary 1

1. Determination of enzyme activity

The extraction of polyphenol oxidase (PPO) and peroxidase (POD) and analysis of activity were performed using the method described by Yi et al. [19] with minor modifications. Briefly, 3 mL sample mixed with 3 mL of solution composed by 4% (w/v) insoluble PVPP, 1 M NaCl and 1% (w/v) Triton X-100 in 0.2 M sodium phosphate with a final pH of 6.5, and centrifuged at 14000 g and 4 °C for 30 min.

PPO activity was assayed spectrophotometrically by adding 3.5 mL of reaction mixture, consisting of 3 mL of 0.07 M pyrocatechol in 0.05 M sodium acetate buffer (pH 6.5), and 0.5 mL of prepared enzyme. The absorbance was measured at 410 nm and 25 °C every 1 s for 3 min.

POD activity was measured at 485 nm and 25 °C every 1 s for 3 min by spectrophotometrically. The reaction started by adding 200 μL of the enzyme extract to 1.5 mL of 0.05 M sodium phosphate buffer (pH 6.5), 200 μL of 1% guaiacol in 0.05 M phosphate buffer (pH 6.5) and 200 μL of 1.5% hydrogen peroxide.

2. Determination of sugar profile

The supernatant was diluted (1:9) in HPLC grade water and filtered through a 0.45 μm nylon membrane for determination of individual sugar by using HPLC (G1315B; Agilent, California, USA) with evaporative light scattering detection (ELSD, G4260B, Agilent, California, USA). Sugar extract (5 μL) was separated on a shodex Asahipak NH2P-50 4E liquid chromatography column (250 mm × 4.6 mm, 5 μm particle size, Agilent, California, USA) coupled to a guard cartridge using an isocratic elution (75% (v/v) acetonitrile/water) at 30 °C. The flow rate was set at 1 mL/min. By comparing the retention time and the peak area with the sugar standard curve, the sugar in the juice was qualitatively and quantified.

3. Determination of organic acid profile

The organic acid profile was analyzed by using a reversed-phase HPLC (1260 Infinity, Agilent, California, USA) equipped with a Prevail Organic Acid column (250 mm × 4.6 mm, 5 μm particle size, Avantor, USA). The isocratic elution was performed using a potassium dihydrogen phosphate buffer (25 mmol/L, pH 2.5) at a flow rate of 0.8 mL/min (25 °C). A UV-DAD detector (G1315B, Agilent, California, USA) at 210 nm was used. Identification and quantification were performed based on retention times and a calibration curve of standard solutions.

4. Determination of aroma compounds

To determine the aromas, an aliquot of 5 mL of samples was transferred to a headspace bottle containing 1.8 g NaCl and 1 μL of Butyl 2-methylbutyrate (100 μL/L, as internal standard), the bottle was sealed by parafilm septum and equilibrated at 40 °C for 5 min. Next, head space was exposed to divinylbenzene/carboxen/polydimethylsiloxane SPME fiber for 10 min at the same temperature with stirring at 300 r/min. Finally, the fiber was obtained and introduced into the GC injector at 250 °C for 5 min. After extraction, the fiber was retracted and immediately inserted into the injection port of Shimadzu GC-2010 Plus gas chromatograph coupled with an GC-MS-QP 2010 Ultra series mass spectrometer, equipped with HP-5 (5% phenyl-polymethylsiloxane, 30 m × 0.25 mm × 0.25 mm, Agilent, California, USA) capillary column to desorb the volatile compounds. A constant column flow of 1.49 mL/min of 99.999% pure helium was used as the carrier gas. The oven temperature was held at 40 °C for 5 min, ramped at the rate of 2 °C/min to 60 °C, held 3 min then ramped to 140 °C at the rate of 5 °C/min, followed by ramping to 250 °C at the rate of 10 °C/min for 5 min [17]. MS was performed with an ion source temperature of 230 °C and an electron ionization energy of 70 eV over the mass range of m/z 35-400 [19]. The identification of aroma compounds was based on a comparison of the mass spectra recorded using the HP-5 column with those in the NIST library. Confirmation of the

identification was completed by comparing the linear retention indices of the compounds with that of an alkane mixture (C_5-C_{25}) and related literature values. Linear retention indices of aroma compounds were calculated using the retention time of n-alkanes obtained under the same GC-MS temperature program, with an injection of 1 μL of n-alkanes. The quantification of aroma compounds was performed using Butyl 2-methylbutyrate as an internal standard. Linear retention index (lri) was calculated according to the following equation[14]:

$$lri = 100n + 100 \times (t_i - t_n)/(t_i - t_n)$$

where n is the number of carbon atoms of compound, t_i is the retention time of the compound, t_n is the retention time of n-alkanes with n carbon atoms, and $t_{(n+1)}$ is the retention time of n-alkanes with n+1 carbon atom.

5. Determination of phenolics

The phenolics in PFP were extracted with 100% methanol buffer. Briefly, 2 mL of samples was extracted with 6 mL methanol (containing 0.1% formic acid), vortexed for 1 min, and incubated at room temperature with ultrasound for 20 min, the extraction mixture was then stored overnight at 4 °C. After centrifugation at 6000 g for 5 min, the supernatants were used for identification. Six independent repetitions were executed for the extraction and subsequent analysis process. And analysed by a Thermo Fisher Ultimate 3000 UHPLC system equipped with a Q-Exactive Orbitrap mass spectrometer (Thermo Fisher Scientific, Bremen, Germany). The optimized program conditions for the LC-MS were as follows: reversed-phase Poroshell HD C18 column (2.1 × 100 mm × 1.8 μm, Agilent, California, USA) was applied and the mobile phases were (A) 0.1% formic acid in acetonitrile and (B) 0.3% formic acid in water at a flow rate of 0.20 mL/min. The column was kept at 35±1 °C and injection volume was 2 μL. Gradient elution was used and the percentage of the water was changed linearly as follows: 0-3 min (95% B), 3-12 min (95-40% B), 12-16 min (40-20% B), 16-20 min (20% B), 20-21 min (20-95% B), 21-25 min (95% B).

The MS conditions were listed as follows: electrospray ionization (ESI) source was set in negative ion mode, scanning range: m/z 100-1500, spray voltage, 3.3 kV; capillary temperature, 320 °C; heater temperature, 350 °C; auxiliary gas flow, 8.0 L/min; sheath gas flow rate, 32.0 L/min; sweep gas, 4.0 L/min; and S-lens RF level, 50%. Identification of compounds was performed by comparing their MS data with those of the corresponding standards, database or those reported in reference. The identified phenolics were quantified or semi-quantified based on their corresponding standard (or at least with similar aglycone) calibration curves.

6. Determination of vitamin C

5 mL of samples was mixed with 20 mL of extraction solution (1% HPO_3 and 0.5% CH_3COOH, pH 2.0), and the mixture was centrifuged at 10,000 g for 30 min at 4 °C. The obtained extract was divided into two parts: one was used for AA analysis and the other was used for vitamin C. Vitamin C, was determined after reduction of DHAA to AA using 2.5 mmol/L TCEP tris (2-carboxyethyl) phosphine in phosphate buffer (pH 3.5). To analyze the AA, phosphate buffer was added to the extract replacing the reducing agent. Both mixtures were centrifuged at 10000 g for 10 min at 4 °C, filtered through a 0.45 μm filter. Separation of the compounds was achieved on a TC-C18 column (250 mm × 4.6 mm, 5 μm particle size, Agilent, California, USA) at 20 °C with a flow rate of 0.8 mL/min. The injection was 25 μL and the detection wavelength was 245 nm. The mobile phase composed of CH_3OH (solvent A) and 1 mmol/L Na_2EDTA and 10 mmol/L CH_3COONH_4 in HPLC-grade H_2O (pH 3.0) (solvent B) and gradient elution program was used as follows: 0-15 min, 5% A, 95% B.

7. Determination of carotenoids

PFP (10 mL) were extracted with 5 mL of solvent (CH_3OH/EtAc/CH_2Cl_2, 25:25:50, v/v/v, containing 0.1% of BHT), stirred for 5 min and placed in an ultrasound bath for 5 min to enhance extraction. The mixture was centrifuged at 17000 g at 4 °C for 5 min. These operations were repeated until colour exhaustion with extracting solvent. The organic phase containing carotenoids was separated and pooled. Finally, the organic phase was concentrated to dryness by rotary evaporation at 30 °C. Before analysis, the extracts were re-dissolved in 1 mL MeOH/MTBE (1:1, v/v) and filtered through 0.45 μm filters. Separation of the compounds was achieved on a reverse phase C30 column (250 × 4.6 mm, 5 μm, YMC Europe GmbH, Dinslaken, Germany) at a flow rate of 0.8 mL/min. Twenty microliter of extract was injected into HPLC (1260 Infinity; Agilent, California, USA) system and the detection wavelength was 450 nm. The mobile phases consisted of H2O/MeOH/MTBE (A/B/C), and following linear gradient program was used: 0 min, 5% A + 90% B + 5% C; 5 min, 0% A + 60% B + 40% C; 20 min, 5% A + 50% B + 45% C; 22 min, 5% A + 50% B + 45% C; 25 min, 5% A + 90% B + 5% C. The chromatograms were run at a wavelength of 450 nm.

Supplementary 2

Table S1. Sensory attributes, definitions and punctuations for the evaluation of yellow passion fruit purée.

Attributes	Definitions	Punctuations	
**Appearance**			
Glossiness	Degree of light reflected from the sample surface	Glossiness better: 9, 8, 7, 6	Reflection ability is poor: 5, 4, 3, 2, 1
Saffron yellow	Saffron yellow color characteristic of passion fruit purée	Saffron yellow pure, uniform: 9, 8, 7, 6	Color is not pure or even: 5, 4, 3, 2, 1
Turbidity	Non-limpid aspect related to the difficulty of light passing through passion fruit purée	Clarification :9, 8, 7, 6	Cloudy:5, 4, 3, 2, 1
Suspended particle	Degree of light reflected on the surface of the sample	No visible particles: 9, 8, 7, 6	Particles are obvious: 5, 4, 3, 2, 1
Aroma			
Natural passion fruit aroma	Characteristic aroma from natural passion fruit purée	Passion fruit fruity: 9, 8, 7, 6	Abnormal smell: 5, 4, 3, 2, 1
Fermented aroma	Characteristic aroma from passion fruit showing signs of early deterioration	No fermentation: 9, 8, 7, 6	Fermentable: 5, 4, 3, 2, 1
Acid aroma	Aroma related to the presence of characteristic organic acids from passion fruit	Pure and moderate acidity: 9, 8, 7, 6	Strong or weak acidity: 5, 4, 3, 2, 1
Sweet aroma	Aroma due to the presence of sucrose and other sugars from passion fruit	Sweetness is pure and moderate: 9, 8, 7, 6	Sweetness strong or weak: 5, 4, 3, 2, 1
Cooked aroma	Characteristic aroma from passion fruit submitted to thermal processing (heat)	No smell of ripeness: 9, 8, 7, 6	Smell of ripeness: 5, 4, 3, 2, 1
Flavor			
Natural passion fruit flavor	Characteristic flavor from natural passion fruit purée	Passion fruit fruity: 9, 8, 7, 6	Abnormal smell: 5, 4, 3, 2, 1
Fermented flavor	Characteristic flavor from passion fruit showing signs of early deterioration	No fermentation: 9, 8, 7, 6	Fermentable: 5, 4, 3, 2, 1

Acid flavor	Flavor related to the presence of characteristic organic acids from passion fruit	Pure and moderate acidity: 9, 8, 7, 6	Strong or weak acidity: 5, 4, 3, 2, 1
Sweet flavor	Flavor due to the presence of sucrose and other sugars from passion fruit	Sweetness is pure and moderate:9, 8, 7, 6	Sweetness strong or weak: 5, 4, 3, 2, 1
Cooked flavor	Characteristic flavor from passion fruit submitted to thermal processing (heat)	No smell of ripeness: 9, 8, 7, 6	Smell of ripeness: 5, 4, 3, 2, 1
		Mouthfeel	
Astringency	Harsh sensation perceived in mouth and tongue characteristic of passion fruit	No stimulation in the mouth or tongue: 9, 8, 7, 6	Mouth and tongue stimulation: 5, 4, 3, 2, 1
Exquisite	Smoothness of the surface of the puree can be felt in the mouth	Smooth texture and even texture: 9, 8, 7, 6	Texture is not fine and the tissue is not even:5, 4, 3, 2, 1

Table S2. Aroma compounds identified in fresh, HPP, PT and HTST passion fruit purée using headspace solid phase microextraction coupled with gas chromatography-mass spectrometry (HS-SPME-GC-MS).

No.	Compounds	CAS	HP-5	Aroma Description
1	2-Pentanone	107-87-9	703	sweet fruity ethereal wine banana woody
2	Ethyl propionate	105-37-3	717.2	sweet fruity rum juicy fruit grape pineapple
3	n-Propyl acetate	109-60-4	720.1	solvent celery fruity fusel raspberry pear
4	Methyl butanoate	623-42-7	727	fruity apple sweet banana pineapple
5	Isoamylol	123-51-3	738	fusel oil alcoholic whiskey fruity banana
6	2-Methylbutanol	137-32-6	740	roasted wine onion fruity fusel alcoholic whiskey
7	1-Pentanol	71-41-0	772	fusel oil sweet balsam
8	Ethyl butanoate	105-54-4	807	fruity juicy fruit pineapple cognac
9	Ethyl crotonate	623-70-1	843	pungent chemical diffusive sweet alliaceous caramel rum
10	Leaf alcohol	928-96-1	857	fresh green cut grass foliage vegetable herbal oily
11	1-Hexanol	111-27-3	870	ethereal fusel oil fruity alcoholic sweet green
12	Propyl butanoate	105-66-8	899	fruity sweet pineapple rancid sweaty
13	2-Heptanol	543-49-7	903	fresh lemon grass herbal sweet floral fruity green
14	Methyl hexoate	106-70-7	925	fruity pineapple ether
15	Ethyl -hydroxybutyrate	5405-41-4	943	fruity green grape tropical apple skin
16	1-Heptanol	111-70-6	970.6	musty leafy violet herbal green sweet woody peony
17	Prenylacetone	110-93-0	985	citrus green musty lemongrass apple
18	β-Myrcene	123-35-3	990	woody, vegetative, citrus, fruity with a tropical mango and slight leafy minty nuances
19	Sulcatol	1569-60-4	993	sweet oily green
20	1-Butyl butyrate	109-21-7	998	fruity banana pineapple green cherry tropical fruit
21	Ethyl hexanoate	123-66-0	1001	sweet fruity pineapple waxy green banana
22	Cis-3-Hexenyl Acetate	3681-71-8	1008	fresh sweet fruity banana apple grassy
23	Hexyl acetate	142-92-7	1015	fruity green apple banana sweet
24	Limonene	138-86-3	1025	citrus herbal terpene camphor
25	(Z)-β-ocimene	13877-91-3	1050	citrus tropical green terpene woody
26	γ-Terpinene	99-85-4	1059	oily woody terpene lemon/lime tropical herbal
27	Linalool oxide	5989-33-3	1074	earthy floral sweet woody
28	1-Octanol	111-87-5	1076	waxy green orange aldehydic rose mushroom
29	Terpinolene	586-62-9	1086	fresh woody sweet pine citrus
30	Propyl hexanoate	626-77-7	1092	sweet fruity juicy pineapple green tropical
31	2-Nonanone	821-55-6	1093	fresh sweet green weedy earthy herbal
32	Linalool	78-70-6	1098	citrus floral sweet bois de rose woody green blueberry
33	Phenylethyl alcohol	60-12-8	1111	floral rose flower rose water
34	Ethyl 3-hydroxyhexanoate	2305-25-1	1130	fruity grape burnt wood hay spicy pineapple cranberry dusty woody
35	Trans-allo-Ocimene	3016-19-1	1143.5	terpene sweet fresh floral
36	Iso-Mentone	491-07-6	1150	minty cool peppermint sweet
37	Hexyl isobutyrate	2349-7-7	1151	green fruity apple pear tart grape ripe berry winey peach
38	Terpinen-4-ol	562-74-3	1175	pepper woody earth musty sweet
39	(E)-3-Hexenyl butyrate	53398-84-8	1185.9	NF

40	Hexyl butanoate	2639-63-6	1193	green sweet fruity apple waxy soapy
41	Ethyl caprylate	106-32-1	1198	fatty fruity winey fermented
42	Decanal	112-31-2	1205	sweet aldehdic waxy orange peel citrus floral
43	Citronellol	106-22-9	1230	floral, rose, sweet, green with fruity citrus nuances
44	Hexyl 2-methylbutyrate	10032-15-2	1238	green waxy fruity apple spicy tropical
45	Theaspirane	36431-72-8	1298	tea herbal green wet tobacco leaf metallic woody spicy
46	Hexyl hexoate	6378-65-0	1386	herbal fresh cut grass vegetable fruity
47	Nerylacetone	3879-26-3	1455	fatty metallic
48	Hexyl octylate	1117-55-1	1584	fruity green waxy berry apple ester
49	Palmitic acid	57-10-3	1958	slightly waxy fatty
50	1-Octadecano	112-92-5	2082	bland
51	Octadecanoic acid	57-11-4	2162	odorless mild fatty waxy

RI = retention index on HP-5 column (Agilent Technologies, California, USA), calculated via triplicated averaged alkanes, and found to be comparable with NIST values (http://webbook.nist.gov/chemistry/name-ser.html). CAS = chemical abstract service number. ID = Identification used as confirmation of compounds per. MS = library match; NF=Not found.

Table S3. LC-QTOF-MS analysis showing the phenolics compounds of fresh yellow passion fruit purée.

No.	molecular formula	identification	retention time (min)	theoretical mass (m/z) $[M-H]^-/[M+H]^+$	measured mass (m/z) $[M-H]^-/[M+H]^+$	mass error (ppm) $[M-H]^-/[M+H]^+$	fragment ion (m/z) $[A-H]^-/[A+H]^+$	Reference
1	$C_{13}H_{16}O_9$	Protocatechuic acid	2.84	153.0185	153.0182	-1.96	108.0204	MS, literature
2	$C_{13}H_{16}O_{10}$	Galloyl-glucoside	3.94	331.0668	331.0668	0.00	125.0230/169.0130	Standard, MS, literature
3	$C_{15}H_{18}O_9$	Caffeic acid hexoside	7.34	341.0881	341.0873	-2.35	135.0438/179.0335	Standard, MS, literature
4	$C1_8H_{24}O_{12}$	Primeveroside salicylic acid	7.83	431.1186	431.1155	-7.19	89.075/71.0124	MS, literature
5	$C_{27}H_{30}O_{16}$	Kaempferol hexosylhexoside	8.30	609.1458	609.1456	-0.33	284.0324/285.0414	Standard, MS, literature
6	$C_{30}H_{26}O_{13}$	Kaempferol-3-coumaroylhexoside	8.60	593.1487	593.1508	3.54	285.0401	Standard, MS, literature
7	$C_{22}H_{26}O_{10}$	Apigenin glycoside derivative	8.74	449.1451	449.1484	7.35	269.0452	MS, literature
8	$C_{21}H_{20}O_{11}$	Astralagin	9.05	447.0930	447.0928	-0.45	285.0395/284.0318/255.029	MS, literature
9	$C_{27}H_{30}O_{15}$	Kaempferol-3-O-glucorhamnoside	9.10	593.1484	593.1508	4.05	284.0318/285.0401/227.0702	Standard, MS, literature
10	$C_{19}H_{14}O_{12}$	Ellagic acid-arabinoside	9.26	433.1136	433.1136	0.00	183.0802/145.0280	MS, literature
11	$C_{21}H_{22}O_{11}$	Eriodictyol-7-O-glucoside	9.33	449.1086	449.1084	-0.45	269.0449	MS, literature
12	$C_{21}H_{24}O_{10}$	Phlorizin	9.34	435.1292	435.1293	0.23	125.0228	Standard, MS, literature
13	$C_{27}H_{30}O_{16}$	Rutin	9.43	609.1458	609.1456	-0.33	255.0296/301.0348/300.0271	Standard, MS, literature
14	$C_9H_{18}O_3$	P-coumaric acid	9.66	163.0407	163.0405	-1.23	93.0331	Standard, MS, literature
15	$C_{21}H_{20}O_{11}$	Luteolin-4"-O-glucoside	9.70	447.0929	447.0928	-0.22	285.0389/151.0024/133.0488	Standard, MS, literature
